# Sea lice loads correlate with the diversity at the Major Histocompatibility Complex ‐related loci in farmed Atlantic salmon, *Salmo salar*

**DOI:** 10.1111/jfd.12986

**Published:** 2019-03-28

**Authors:** Rebecca Jane Pawluk, Carlos Garcia de Leaniz, Sofia Consuegra

**Affiliations:** ^1^ Swansea University Swansea UK

**Keywords:** ectoparasites, genotype, *Lepeophtheirus salmonis*, Major Histocompatibility Complex, salmonid pathogen

Intensively farmed fish often display reduced genetic diversity compared to wild populations due to mating among close relatives and artificial selection for commercially important traits (Kijas et al., 2016; Roberge, Einum, Guderley, & Bernatchez, 2006), which can make them more susceptible to parasites. Farmed Atlantic salmon are frequently infected with sea lice (*Lepeophtheirus salmonis*), a parasitic copepod that causes high mortalities and economic losses to salmon farming (Costello, 2009), and whose impact could be exacerbated by reduced genetic diversity, as seen in other species (Blanchet, Rey, Berthier, Lek, & Loot, 2009). Yet, despite the large numbers of farmed salmon and the high incidence of sea lice, most studies have focused on the genetic diversity of the parasite (Todd, Walker, Ritchie, Graves, & Walker, 2004) or on the identification of markers for salmon resistance (Jones, Lockyer, Verspoor, Secombes, & Noble, 2002), while less attention has been paid to the relationship between parasite number and salmon genetic diversity.

Resistance to the parasite *Anisakis* sp. in Atlantic salmon has previously been linked to variation at the major histocompatibility complex (MHC; Consuegra & Garcia de Leaniz, 2008). Furthermore, previous studies have indicated a link between variation at the MHC and sea lice abundance (Gharbi et al., 2009; Glover et al., 2007). However, relationships between genetic diversity at neutral loci and host fitness have also been identified, for example, in roe deer and Egyptian vultures (Agudo et al., 2012), and thus, it appears to be important to consider functional and neutral markers when inferring host resistance.

Here, we examined the association between sea lice abundance and genetic diversity at 15 microsatellite loci (including neutral markers and two MHC‐linked loci; Supporting Information Table S2) in 54 Scottish Atlantic salmon sampled in sea cages after 1 month (site SU) or 13 months at sea (site SB). Total genomic DNA was extracted from adipose fin clips and amplified as described in Ellis et al. (2011) and Supporting Information Table S1. To quantify genetic diversity, we estimated the number of alleles (*N*_a_), the observed (*H*_o_) and expected (*H*_e_) heterozygosity, the effective population size (*N*_e_) and pairwise relatedness (Supporting Information Tables S2–S5). Individual homozygosity by locus (HL) was calculated using Cernicalin (Aparicio, Ortego, & Cordero, 2006), whereby a value of 0 indicates complete heterozygosity and 1 indicates complete homozygosity. We used generalized linear models with a poisson link in R 3.4.0 (R Core Team, 2014) to examine individual variation in sea lice abundance as a function of body size, site and individual homozygosity by loci and immune‐related loci, correcting for overdispersion.

Measures of genetic diversity and relatedness were moderate and statistically similar between sites (Table 1). Estimates of effective population size (*N*_e_) were approximately twice as high as those reported for Norwegian farm strains (Karlsson, Moen, & Hindar, 2010) and some marginal wild populations (Consuegra, Verspoor, Knox, & Garcıa de Leaniz, 2005), but were within the range found for Finnish populations under supportive breeding (Säisä, Koljonen, & Tähtinen, 2003). Although observed *N*_e_ estimates were below the 500 value recommended for maintaining long‐term evolutionary potential (Frankham, Bradshaw, & Brook, 2014), they were high enough to avoid inbreeding in the short term (*N*_e_ > 50). Pairwise relatedness was lower than that found for other farm salmon populations (Karlsson et al., 2010), indicating relatively low levels of inbreeding.

**Table 1:** Genetic diversity, body size and condition, and sea lice abundance in 54 farmed Atlantic salmon (*Salmo salar*) sampled during two stages in the marine production cycle (site SU: c. 1 month post‐marine deployment; site SB: c. 13 months post‐marine deployment)

Parameter	Site SU (c. 1 month post‐deployment)	Site SB (c. 13 months post‐deployment)
Genetic diversity		
*N*	27.00	27.00
*N*_a_	8.80	8.93
*I*	1.76	1.74
*H*_o_	0.63	0.63
*H*_e_	0.76	0.77
*F*	0.16	0.15
Effective population size (*N*_e_)		
LDM method	108.1 (95% CI: 57.6–515.6)	102.8 (95% CI: 61.9–261.1)
MCM method	117.6 (95% CI: 0.1–590.2)	186.6 (95% CI: 0.2–936.8)
Relatedness		
LRM method	−0.019 (*SD*: 0.044)	−0.019 (*SD*: 0.046)
QGM method	−0.038 (*SD*: 0.038)	−0.038 (*SD*: 0.038)
Homozygosity by locus (HL)		
All loci	0.32 (*SE*: 0.02)	0.33 (*SE*: 0.03)
MHC‐related loci	0.51 (*SE*: 0.07)	0.61 (*SE*: 0.08)
Fish size and condition		
Standard length (cm)	22.28 (*SE*: 0.42)	58.04 (*SE*: 1.35)
Weight (kg)	0.14 (*SE*: 0.01)	2.25 (*SE*: 0.16)
Body condition (CF)	1.27 (*SE*: 0.05)	1.16 (*SE*: 0.08)
Sea lice		
Abundance (No/fish)	2.63 (*SE*: 0.47)	10.15 (*SE*: 1.24)
Prevalence (%)	85.2 (95% CI: 66.3–95.8)	96.3 (95% CI: 81.0–99.9)

Sea lice abundance varied greatly among farmed salmon (range: 0–28), but was unrelated to fish size, or to homozygosity by neutral loci (HL), once the effects of site were accounted for (Supporting Information Tables S6 and S7, Figure 1a). Instead, sea lice load was influenced by homozygosity at MHC‐linked loci (HLmhc, *t *= −2.838, *p* = 0.007), as well as by site (*t *= −3.33, *p* = 0.002) and the interactions between HLmhc and length (*t* = 2.67, *p* = 0.01) and between HLmhc and site (*t* = 2.751, *p* = 0.008; Supporting Information Table S6). Increased homozygosity at MHC‐linked loci resulted in fewer sea lice, particularly for 13‐month‐old post‐smolts (site SB; Figure 1b). Previous work had indicated a link between MHC markers and sea lice resistance (Gharbi et al., 2009; Glover et al., 2007). We found a potentially selective advantage for MHC (but not neutral markers) homozygous individuals with respect to parasite loads, suggesting that MHC diversity could be more important than genome‐wide genetic variation in the resistance to this particular parasite. MHC homozygosity advantage has been seen in other species (Wedekind, Walker, & Little, 2005), and our results further confirm the role for MHC on sea lice resistance in Atlantic salmon. Given the threat posed by sea lice to salmon farming, we suggest that MHC variation should be considered in selective breeding programmes.

**Figure 1:**
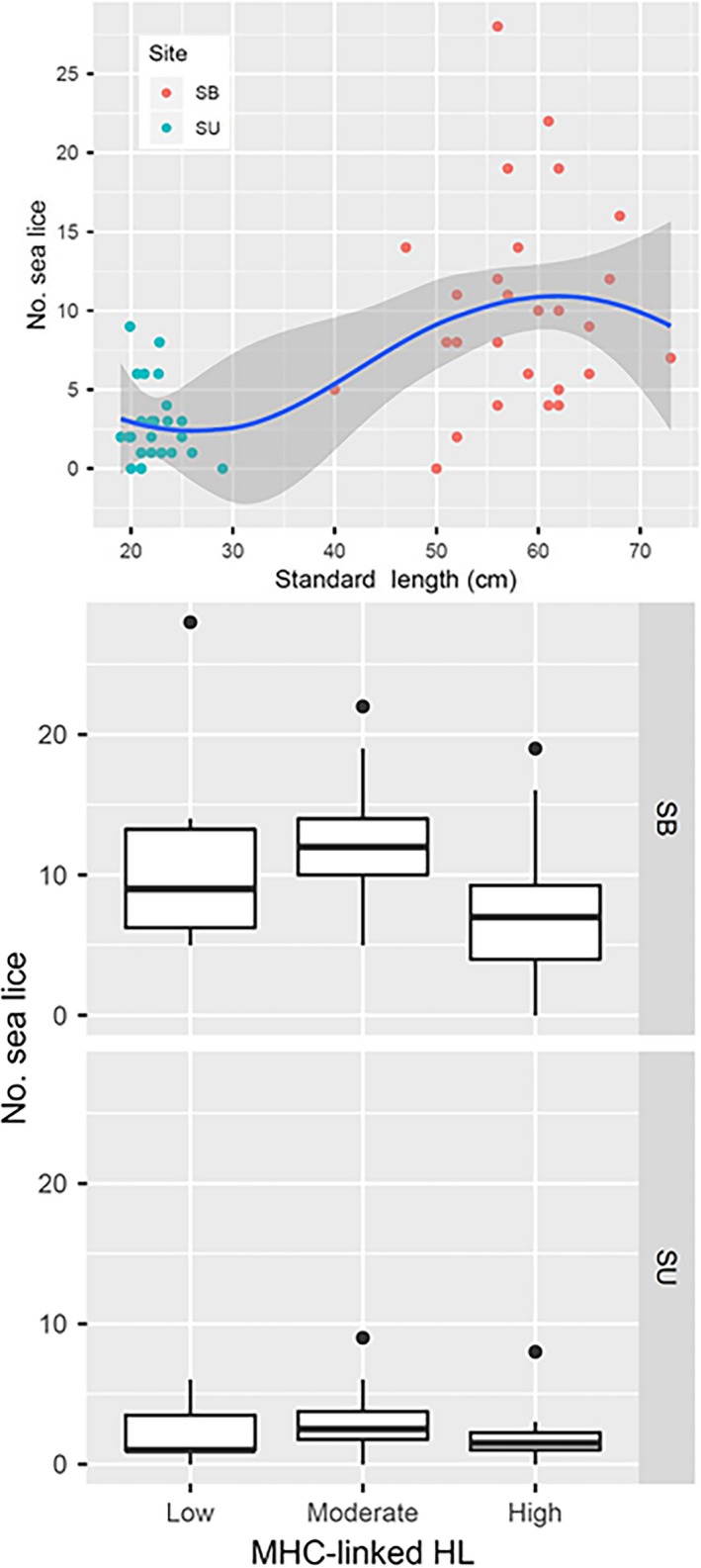
Influence of (a) standard length (cm) and (b) homozygosity by MHC‐linked loci on sea lice abundance (No. sea lice/fish) on farmed Atlantic salmon at two stages during the marine production cycle (site SU: 1‐month‐old post‐smolts; site SB: 13‐month‐old post‐smolts) [Colour figure can be viewed at http://wileyonlinelibrary.com]

## CONFLICT OF INTEREST

The authors declare that they have no conflict of interest.

## Data Availability

All data have been stored in Figshare (https://figshare.com/s/a77eea0693862c4d5ddf).
