# Supplementary material for: Sea lice loads correlate with the diversity at the Major Histocompatibility Complex ‐related loci in farmed Atlantic salmon, Salmo salar
Source: J Fish Dis. 2019 Mar 28;42(7):1091–3. doi: 10.1111/jfd.12986 (PMC6850134; doi:10.1111/jfd.12986)
Supplement: Supplementary file 1 [file JFD-42-1091-s001.docx]

**Supplementary material for****:** **Sea lice loads correlate with the diversity at the MHC-related loci in farmed Atlantic salmon, *Salmo salar***

**Table S1.** PCR conditions used for genotyping 60 Atlantic salmon samples at 15 microsatellites.

| ***Phase*** | ***Time*** | ***Unit*** | ***Temp*** | ***unit*** | ***Cycles*** |
| --- | --- | --- | --- | --- | --- |
| *Initial activation* | 15 | min | 95 | C | 1 |
| *Denaturation* | 30 | s | 94 | C |  |
| *Annealing* | 90 | s | 56 | C |  |
| *Extension* | 90 | s | 72 | C | 8 |
| *Denaturation* | 30 | s | 94 | C |  |
| *Annealing* | 90 | s | 56 | C |  |
| *Extension* | 90 | s | 72 | C | 24 |
| *Final extension* | 10 | min | 72 | C | 1 |

**Table S2.** Genetic diversity (at 15 microsatellite loci) for 54 sea louse infected Atlantic salmon (*Salmo salar*) at two farm cages (SU and SB).

| **Pop** | **Locus** | **N** | **Na** | **Ne** | **I** | **Ho** | **He** | **F** |
| --- | --- | --- | --- | --- | --- | --- | --- | --- |
| **SU** | **SSsp2210** | 27 | 10.000 | 4.365 | 1.815 | 0.333 | 0.771 | 0.568 |
|  | **Ssa202** | 27 | 13.000 | 6.231 | 2.144 | 0.815 | 0.840 | 0.029 |
|  | **SSspG7** | 27 | 11.000 | 6.943 | 2.150 | 0.741 | 0.856 | 0.135 |
|  | **Sp2201** | 27 | 11.000 | 5.695 | 2.016 | 0.852 | 0.824 | -0.033 |
|  | **SsaD144** | 27 | 14.000 | 9.529 | 2.434 | 0.630 | 0.895 | 0.297 |
|  | **Sasa-UBA** | 27 | 9.000 | 5.608 | 1.895 | 0.630 | 0.822 | 0.234 |
|  | **Sp1605** | 27 | 7.000 | 4.352 | 1.711 | 0.815 | 0.770 | -0.058 |
|  | **SsaF43** | 27 | 1.000 | 1.000 | 0.000 | 0.000 | 0.000 | #N/A |
|  | **Sp2216** | 27 | 11.000 | 6.845 | 2.134 | 0.778 | 0.854 | 0.089 |
|  | **Ssa197** | 27 | 9.000 | 6.231 | 1.963 | 0.852 | 0.840 | -0.015 |
|  | **SSsp3016** | 27 | 8.000 | 3.767 | 1.599 | 0.741 | 0.735 | -0.008 |
|  | **SSOSL85** | 27 | 9.000 | 7.218 | 2.068 | 0.815 | 0.861 | 0.054 |
|  | **Sasa-DAA** | 27 | 6.000 | 4.571 | 1.620 | 0.333 | 0.781 | 0.573 |
|  | **Ssa289** | 27 | 5.000 | 1.813 | 0.892 | 0.296 | 0.449 | 0.339 |
|  | **Ssa171** | 27 | 8.000 | 6.509 | 1.942 | 0.852 | 0.846 | -0.006 |
|  |  |  |  |  |  |  |  |  |
| **SB** | **SSsp2210** | 27 | 11.000 | 5.226 | 1.950 | 0.370 | 0.809 | 0.542 |
|  | **Ssa202** | 27 | 6.000 | 3.247 | 1.382 | 0.741 | 0.692 | -0.070 |
|  | **SSspG7** | 27 | 11.000 | 6.423 | 2.040 | 0.667 | 0.844 | 0.210 |
|  | **Sp2201** | 27 | 13.000 | 7.755 | 2.319 | 0.889 | 0.871 | -0.020 |
|  | **SsaD144** | 27 | 12.000 | 6.719 | 2.145 | 0.556 | 0.851 | 0.347 |
|  | **Sasa-UBA** | 27 | 10.000 | 4.765 | 1.815 | 0.370 | 0.790 | 0.531 |
|  | **Sp1605** | 27 | 8.000 | 5.673 | 1.872 | 0.852 | 0.824 | -0.034 |
|  | **SsaF43** | 27 | 2.000 | 1.117 | 0.215 | 0.111 | 0.105 | -0.059 |
|  | **Sp2216** | 27 | 10.000 | 6.204 | 2.006 | 0.741 | 0.839 | 0.117 |
|  | **Ssa197** | 27 | 12.000 | 6.976 | 2.166 | 0.741 | 0.857 | 0.135 |
|  | **SSsp3016** | 27 | 9.000 | 3.321 | 1.544 | 0.630 | 0.699 | 0.099 |
|  | **SSOSL85** | 27 | 10.000 | 7.218 | 2.102 | 0.926 | 0.861 | -0.075 |
|  | **Sasa-DAA** | 27 | 6.000 | 3.609 | 1.420 | 0.407 | 0.723 | 0.436 |
|  | **Ssa289** | 27 | 6.000 | 3.513 | 1.383 | 0.593 | 0.715 | 0.172 |
|  | **Ssa171** | 27 | 8.000 | 5.010 | 1.757 | 0.815 | 0.800 | -0.018 |

**Table S3**. Hardy Weinberg equilibrium values and population summary tables for all loci in two separate Atlantic salmon populations (SU and SB). Significant P values (after Bonferroni adjustment) are indicated by italics.

| **Population** | **locus** | **P-val** | **S.E.** | **W&C** | **R&H** |
| --- | --- | --- | --- | --- | --- |
|  | SSsp2210 | *0* | 0 | 0.58 | 0.475 |
| **SU** | Ssa202 | 0.041 | 0.011 | 0.048 | 0.031 |
|  | SSspG7 | 0.115 | 0.015 | 0.153 | 0.225 |
|  | Sp2201 | 0.45 | 0.026 | -0.014 | -0.001 |
|  | SsaD144 | *0* | 0 | 0.314 | 0.219 |
|  | Sasa-UBA | *0* | 0 | 0.252 | 0.298 |
|  | Sp1605 | 0.64 | 0.013 | -0.039 | -0.004 |
|  | SsaF43 | No information |  | . |  |
|  | Sp2216 | 0.225 | 0.022 | 0.108 | 0.059 |
|  | Ssa197 | 0.064 | 0.008 | 0.004 | 0.162 |
|  | SSsp3016 | 0.553 | 0.017 | 0.011 | 0.02 |
|  | SSOSL85 | 0.037 | 0.006 | 0.073 | 0.03 |
|  | Sasa-DAA | *0* | 0 | 0.586 | 0.77 |
|  | Ssa289 | 0.017 | 0.003 | 0.356 | 0.204 |
|  | Ssa171 | 0.337 | 0.014 | 0.012 | -0.009 |
|  |  |  |  |  |  |
| **SB** | SSsp2210 | *0* | 0 | 0.555 | 0.301 |
|  | Ssa202 | *0.002* | 0.001 | -0.052 | 0.186 |
|  | SSspG7 | *0.002* | 0.001 | 0.228 | 0.191 |
|  | Sp2201 | 0.602 | 0.027 | -0.002 | 0.008 |
|  | SsaD144 | 0.005 | 0.004 | 0.364 | 0.29 |
|  | Sasa-UBA | *0* | 0 | 0.545 | 0.308 |
|  | Sp1605 | 0.786 | 0.012 | -0.015 | -0.026 |
|  | SsaF43 | 1 | 0 | -0.04 | -0.041 |
|  | Sp2216 | 0.549 | 0.022 | 0.136 | 0.111 |
|  | Ssa197 | 0.114 | 0.017 | 0.154 | 0.066 |
|  | SSsp3016 | 0.066 | 0.011 | 0.118 | 0.054 |
|  | SSOSL85 | 0.014 | 0.004 | -0.056 | 0.044 |
|  | Sasa-DAA | *0.002* | 0.001 | 0.452 | 0.3 |
|  | Ssa289 | 0.011 | 0.003 | 0.19 | 0.074 |
|  | Ssa171 | 0.044 | 0.007 | 0.001 | -0.013 |
|  |  |  |  |  |  |
|  | **SU** | **SB** | **Both Sites** |  |  |
| **ChiSq** | Infinity | Infinity | Infinity |  |  |
| **DF** | 28 | 30 | 46 |  |  |
| **Prob** | High.sign | High.sign | High.sign |  |  |

**Table S4.** Linkage disequilibrium for all loci for both sites

| **Pop** | **Locus#1** | **Locus#2** | **P-Value** | **S.E.** | **Switches** |
| --- | --- | --- | --- | --- | --- |
| SU | SSsp2210 | Ssa202 | 0.28392 | 0.037924 | 1343 |
| SU | SSsp2210 | SSspG7 | 1 | 0 | 1046 |
| SU | Ssa202 | SSspG7 | 0.3587 | 0.043567 | 634 |
| SU | SSsp2210 | Sp2201 | 0.44062 | 0.039061 | 1379 |
| SU | Ssa202 | Sp2201 | 0.27101 | 0.039244 | 797 |
| SU | SSspG7 | Sp2201 | 1 | 0 | 625 |
| SU | SSsp2210 | SsaD144 | 0.58457 | 0.040831 | 881 |
| SU | Ssa202 | SsaD144 | 0.58579 | 0.046605 | 491 |
| SU | SSspG7 | SsaD144 | 0.22909 | 0.038787 | 385 |
| SU | Sp2201 | SsaD144 | 0.24077 | 0.03928 | 493 |
| SU | SSsp2210 | Sasa-UBA | 0.01115 | 0.006204 | 1784 |
| SU | Ssa202 | Sasa-UBA | 0.87231 | 0.027663 | 1068 |
| SU | SSspG7 | Sasa-UBA | 1 | 0 | 806 |
| SU | Sp2201 | Sasa-UBA | 0.16462 | 0.031064 | 1103 |
| SU | SsaD144 | Sasa-UBA | 0.55202 | 0.045597 | 666 |
| SU | SSsp2210 | Sp1605 | 1 | 0 | 1776 |
| SU | Ssa202 | Sp1605 | 1 | 0 | 1110 |
| SU | SSspG7 | Sp1605 | 1 | 0 | 876 |
| SU | Sp2201 | Sp1605 | 1 | 0 | 1087 |
| SU | SsaD144 | Sp1605 | 0.35963 | 0.044565 | 692 |
| SU | Sasa-UBA | Sp1605 | 0.54651 | 0.037858 | 1505 |
| SU | SSsp2210 | SsaF43 | # | # |  |
| SU | Ssa202 | SsaF43 | # | # |  |
| SU | SSspG7 | SsaF43 | # | # |  |
| SU | Sp2201 | SsaF43 | # | # |  |
| SU | SsaD144 | SsaF43 | # | # |  |
| SU | Sasa-UBA | SsaF43 | # | # |  |
| SU | Sp1605 | SsaF43 | # | # |  |
| SU | SSsp2210 | Sp2216 | 0.74311 | 0.033127 | 1068 |
| SU | Ssa202 | Sp2216 | 1 | 0 | 606 |
| SU | SSspG7 | Sp2216 | 0.35076 | 0.045566 | 418 |
| SU | Sp2201 | Sp2216 | 1 | 0 | 588 |
| SU | SsaD144 | Sp2216 | 1 | 0 | 390 |
| SU | Sasa-UBA | Sp2216 | 0.16519 | 0.031948 | 813 |
| SU | Sp1605 | Sp2216 | 1 | 0 | 826 |
| SU | SsaF43 | Sp2216 | # | # |  |
| SU | SSsp2210 | Ssa197 | 0.9372 | 0.018592 | 1260 |
| SU | Ssa202 | Ssa197 | 0.69333 | 0.039155 | 703 |
| SU | SSspG7 | Ssa197 | 1 | 0 | 600 |
| SU | Sp2201 | Ssa197 | 1 | 0 | 744 |
| SU | SsaD144 | Ssa197 | 1 | 0 | 487 |
| SU | Sasa-UBA | Ssa197 | 0.52892 | 0.040967 | 1014 |
| SU | Sp1605 | Ssa197 | 0.83289 | 0.027956 | 1064 |
| SU | SsaF43 | Ssa197 | # | # |  |
| SU | Sp2216 | Ssa197 | 0.38601 | 0.043889 | 597 |
| SU | SSsp2210 | SSsp3016 | 0.85959 | 0.024111 | 1983 |
| SU | Ssa202 | SSsp3016 | 0.04698 | 0.014861 | 1252 |
| SU | SSspG7 | SSsp3016 | 0.01204 | 0.007988 | 902 |
| SU | Sp2201 | SSsp3016 | 0.38263 | 0.039402 | 1223 |
| SU | SsaD144 | SSsp3016 | 1 | 0 | 791 |
| SU | Sasa-UBA | SSsp3016 | 0.46635 | 0.040453 | 1580 |
| SU | Sp1605 | SSsp3016 | 0.2868 | 0.03376 | 1603 |
| SU | SsaF43 | SSsp3016 | # | # |  |
| SU | Sp2216 | SSsp3016 | 1 | 0 | 934 |
| SU | Ssa197 | SSsp3016 | 1 | 0 | 1175 |
| SU | SSsp2210 | SSOSL85 | 0.44088 | 0.038882 | 1550 |
| SU | Ssa202 | SSOSL85 | 0.00192 | 0.00192 | 860 |
| SU | SSspG7 | SSOSL85 | 1 | 0 | 649 |
| SU | Sp2201 | SSOSL85 | 0.31417 | 0.040328 | 939 |
| SU | SsaD144 | SSOSL85 | 1 | 0 | 552 |
| SU | Sasa-UBA | SSOSL85 | 0.77464 | 0.03358 | 1235 |
| SU | Sp1605 | SSOSL85 | 0.52207 | 0.040726 | 1251 |
| SU | SsaF43 | SSOSL85 | # | # |  |
| SU | Sp2216 | SSOSL85 | 0.22558 | 0.037575 | 691 |
| SU | Ssa197 | SSOSL85 | 0.03842 | 0.017158 | 901 |
| SU | SSsp3016 | SSOSL85 | 0.6949 | 0.036596 | 1492 |
| SU | SSsp2210 | Sasa-DAA | 0.36216 | 0.024607 | 4711 |
| SU | Ssa202 | Sasa-DAA | 0.88599 | 0.016983 | 3167 |
| SU | SSspG7 | Sasa-DAA | 0.67405 | 0.030636 | 2454 |
| SU | Sp2201 | Sasa-DAA | 0.11653 | 0.018814 | 3263 |
| SU | SsaD144 | Sasa-DAA | 0.6349 | 0.03472 | 2111 |
| SU | Sasa-UBA | Sasa-DAA | 0.33704 | 0.027017 | 4089 |
| SU | Sp1605 | Sasa-DAA | 0.71327 | 0.02386 | 4124 |
| SU | SsaF43 | Sasa-DAA | # | # |  |
| SU | Sp2216 | Sasa-DAA | 1 | 0 | 2549 |
| SU | Ssa197 | Sasa-DAA | 0.96994 | 0.009079 | 2924 |
| SU | SSsp3016 | Sasa-DAA | 0.92229 | 0.01076 | 4305 |
| SU | SSOSL85 | Sasa-DAA | 1 | 0 | 3668 |
| SU | SSsp2210 | Ssa289 | 0.40378 | 0.029066 | 3205 |
| SU | Ssa202 | Ssa289 | 0.62737 | 0.033872 | 2334 |
| SU | SSspG7 | Ssa289 | 0.18703 | 0.028315 | 2008 |
| SU | Sp2201 | Ssa289 | 0.95116 | 0.01014 | 2411 |
| SU | SsaD144 | Ssa289 | 0.94337 | 0.015323 | 1819 |
| SU | Sasa-UBA | Ssa289 | 0.44319 | 0.032634 | 2814 |
| SU | Sp1605 | Ssa289 | 0.91534 | 0.013534 | 3012 |
| SU | SsaF43 | Ssa289 | # | # |  |
| SU | Sp2216 | Ssa289 | 0.85878 | 0.024055 | 1987 |
| SU | Ssa197 | Ssa289 | 0.61006 | 0.033277 | 2288 |
| SU | SSsp3016 | Ssa289 | 0.57404 | 0.030187 | 3011 |
| SU | SSOSL85 | Ssa289 | 0.78566 | 0.020502 | 2780 |
| SU | Sasa-DAA | Ssa289 | 0.20258 | 0.014958 | 6640 |
| SU | SSsp2210 | Ssa171 | 0.31151 | 0.036402 | 1415 |
| SU | Ssa202 | Ssa171 | 0.64607 | 0.041194 | 797 |
| SU | SSspG7 | Ssa171 | 0.37164 | 0.044329 | 639 |
| SU | Sp2201 | Ssa171 | 0.05564 | 0.01755 | 794 |
| SU | SsaD144 | Ssa171 | 1 | 0 | 516 |
| SU | Sasa-UBA | Ssa171 | 0.6335 | 0.039226 | 1125 |
| SU | Sp1605 | Ssa171 | 0.62152 | 0.041559 | 1123 |
| SU | SsaF43 | Ssa171 | # | # |  |
| SU | Sp2216 | Ssa171 | 0.00966 | 0.006805 | 587 |
| SU | Ssa197 | Ssa171 | 1 | 0 | 753 |
| SU | SSsp3016 | Ssa171 | 0.06862 | 0.019853 | 1204 |
| SU | SSOSL85 | Ssa171 | 1 | 0 | 974 |
| SU | Sasa-DAA | Ssa171 | 0.37527 | 0.028808 | 3225 |
| SU | Ssa289 | Ssa171 | 0.56903 | 0.029994 | 2453 |
| SB | SSsp2210 | Ssa202 | 0.17821 | 0.024107 | 2468 |
| SB | SSsp2210 | SSspG7 | 0.8842 | 0.023525 | 1288 |
| SB | Ssa202 | SSspG7 | 0.86107 | 0.021395 | 2209 |
| SB | SSsp2210 | Sp2201 | 0.6678 | 0.041131 | 836 |
| SB | Ssa202 | Sp2201 | 0.46545 | 0.037551 | 1518 |
| SB | SSspG7 | Sp2201 | 1 | 0 | 642 |
| SB | SSsp2210 | SsaD144 | 0.5685 | 0.041529 | 1006 |
| SB | Ssa202 | SsaD144 | 0.05144 | 0.013151 | 1807 |
| SB | SSspG7 | SsaD144 | 1 | 0 | 887 |
| SB | Sp2201 | SsaD144 | 0.34732 | 0.04538 | 481 |
| SB | SSsp2210 | Sasa-UBA | 0.07935 | 0.022954 | 1361 |
| SB | Ssa202 | Sasa-UBA | 0.76848 | 0.026585 | 2317 |
| SB | SSspG7 | Sasa-UBA | 0.74693 | 0.035104 | 1210 |
| SB | Sp2201 | Sasa-UBA | 0.54439 | 0.043117 | 768 |
| SB | SsaD144 | Sasa-UBA | 1 | 0 | 898 |
| SB | SSsp2210 | Sp1605 | 0.86408 | 0.024679 | 1210 |
| SB | Ssa202 | Sp1605 | 0.95983 | 0.011106 | 2015 |
| SB | SSspG7 | Sp1605 | 0.55745 | 0.04441 | 910 |
| SB | Sp2201 | Sp1605 | 1 | 0 | 602 |
| SB | SsaD144 | Sp1605 | 1 | 0 | 685 |
| SB | Sasa-UBA | Sp1605 | 1 | 0 | 1014 |
| SB | SSsp2210 | SsaF43 | 0.73794 | 0.009245 | 11905 |
| SB | Ssa202 | SsaF43 | 1 | 0 | 15002 |
| SB | SSspG7 | SsaF43 | 0.89916 | 0.004914 | 12587 |
| SB | Sp2201 | SsaF43 | 0.93504 | 0.005157 | 10897 |
| SB | SsaD144 | SsaF43 | 0.47474 | 0.010769 | 11318 |
| SB | Sasa-UBA | SsaF43 | 0.81632 | 0.007265 | 11892 |
| SB | Sp1605 | SsaF43 | 0.26322 | 0.009907 | 12157 |
| SB | SSsp2210 | Sp2216 | 0.58635 | 0.041459 | 883 |
| SB | Ssa202 | Sp2216 | 0.16795 | 0.02733 | 1507 |
| SB | SSspG7 | Sp2216 | 1 | 0 | 697 |
| SB | Sp2201 | Sp2216 | 1 | 0 | 413 |
| SB | SsaD144 | Sp2216 | 1 | 0 | 529 |
| SB | Sasa-UBA | Sp2216 | 0.44431 | 0.044781 | 879 |
| SB | Sp1605 | Sp2216 | 0.36127 | 0.044536 | 604 |
| SB | SsaF43 | Sp2216 | 0.15701 | 0.006776 | 10810 |
| SB | SSsp2210 | Ssa197 | 0.66022 | 0.04162 | 754 |
| SB | Ssa202 | Ssa197 | 0.52752 | 0.039638 | 1543 |
| SB | SSspG7 | Ssa197 | 0.18228 | 0.034414 | 636 |
| SB | Sp2201 | Ssa197 | 1 | 0 | 386 |
| SB | SsaD144 | Ssa197 | 1 | 0 | 475 |
| SB | Sasa-UBA | Ssa197 | 0.15876 | 0.030709 | 762 |
| SB | Sp1605 | Ssa197 | 1 | 0 | 552 |
| SB | SsaF43 | Ssa197 | 0.50999 | 0.017245 | 10378 |
| SB | Sp2216 | Ssa197 | 1 | 0 | 397 |
| SB | SSsp2210 | SSsp3016 | 0.32499 | 0.035237 | 2032 |
| SB | Ssa202 | SSsp3016 | 0.95155 | 0.011461 | 3229 |
| SB | SSspG7 | SSsp3016 | 0.05207 | 0.015409 | 1747 |
| SB | Sp2201 | SSsp3016 | 1 | 0 | 1269 |
| SB | SsaD144 | SSsp3016 | 0.41289 | 0.038543 | 1499 |
| SB | Sasa-UBA | SSsp3016 | 0.39448 | 0.033736 | 2024 |
| SB | Sp1605 | SSsp3016 | 0.40903 | 0.037039 | 1738 |
| SB | SsaF43 | SSsp3016 | 0.2437 | 0.006809 | 14614 |
| SB | Sp2216 | SSsp3016 | 0.64285 | 0.038753 | 1268 |
| SB | Ssa197 | SSsp3016 | 0.69724 | 0.036662 | 1141 |
| SB | SSsp2210 | SSOSL85 | 0.79695 | 0.033099 | 1226 |
| SB | Ssa202 | SSOSL85 | 0.23469 | 0.028694 | 2056 |
| SB | SSspG7 | SSOSL85 | 1 | 0 | 980 |
| SB | Sp2201 | SSOSL85 | 1 | 0 | 611 |
| SB | SsaD144 | SSOSL85 | 0.62354 | 0.041164 | 734 |
| SB | Sasa-UBA | SSOSL85 | 0.15688 | 0.03027 | 1154 |
| SB | Sp1605 | SSOSL85 | 0.39519 | 0.041713 | 852 |
| SB | SsaF43 | SSOSL85 | 0.56732 | 0.010905 | 11425 |
| SB | Sp2216 | SSOSL85 | 0.31656 | 0.041018 | 682 |
| SB | Ssa197 | SSOSL85 | 0.44178 | 0.043654 | 616 |
| SB | SSsp3016 | SSOSL85 | 0.94357 | 0.015409 | 1711 |
| SB | SSsp2210 | Sasa-DAA | 0.74237 | 0.030813 | 2167 |
| SB | Ssa202 | Sasa-DAA | 0.49085 | 0.031743 | 3232 |
| SB | SSspG7 | Sasa-DAA | 0.04573 | 0.012237 | 1872 |
| SB | Sp2201 | Sasa-DAA | 0.49276 | 0.042806 | 1216 |
| SB | SsaD144 | Sasa-DAA | 0.59911 | 0.037263 | 1390 |
| SB | Sasa-UBA | Sasa-DAA | 0.01193 | 0.006424 | 1982 |
| SB | Sp1605 | Sasa-DAA | 0.76707 | 0.029755 | 1699 |
| SB | SsaF43 | Sasa-DAA | 0.29032 | 0.006905 | 14008 |
| SB | Sp2216 | Sasa-DAA | 0.58115 | 0.043198 | 1248 |
| SB | Ssa197 | Sasa-DAA | 0.13525 | 0.023855 | 1175 |
| SB | SSsp3016 | Sasa-DAA | 0.25226 | 0.025248 | 2851 |
| SB | SSOSL85 | Sasa-DAA | 0.16661 | 0.025938 | 1705 |
| SB | SSsp2210 | Ssa289 | 0.52861 | 0.032585 | 2817 |
| SB | Ssa202 | Ssa289 | 0.78207 | 0.022258 | 4296 |
| SB | SSspG7 | Ssa289 | 1 | 0 | 2651 |
| SB | Sp2201 | Ssa289 | 1 | 0 | 1759 |
| SB | SsaD144 | Ssa289 | 0.93095 | 0.016907 | 2072 |
| SB | Sasa-UBA | Ssa289 | 0.49056 | 0.032334 | 2820 |
| SB | Sp1605 | Ssa289 | 0.85153 | 0.023599 | 2314 |
| SB | SsaF43 | Ssa289 | 0.44805 | 0.007208 | 16359 |
| SB | Sp2216 | Ssa289 | 0.05855 | 0.016035 | 1893 |
| SB | Ssa197 | Ssa289 | 0.73845 | 0.030369 | 1704 |
| SB | SSsp3016 | Ssa289 | 0.99618 | 0.001706 | 3879 |
| SB | SSOSL85 | Ssa289 | 0.40494 | 0.030857 | 2396 |
| SB | Sasa-DAA | Ssa289 | 0.48234 | 0.02657 | 3824 |
| SB | SSsp2210 | Ssa171 | 0.99139 | 0.005522 | 1906 |
| SB | Ssa202 | Ssa171 | 0.76666 | 0.025476 | 3139 |
| SB | SSspG7 | Ssa171 | 1 | 0 | 1655 |
| SB | Sp2201 | Ssa171 | 0.37895 | 0.041693 | 1079 |
| SB | SsaD144 | Ssa171 | 0.7619 | 0.032795 | 1320 |
| SB | Sasa-UBA | Ssa171 | 0.36127 | 0.032892 | 1830 |
| SB | Sp1605 | Ssa171 | 0.73278 | 0.035983 | 1490 |
| SB | SsaF43 | Ssa171 | 0.2505 | 0.007656 | 14531 |
| SB | Sp2216 | Ssa171 | 0.5622 | 0.042354 | 1081 |
| SB | Ssa197 | Ssa171 | 0.63765 | 0.041815 | 1011 |
| SB | SSsp3016 | Ssa171 | 0.2529 | 0.024484 | 2730 |
| SB | SSOSL85 | Ssa171 | 0.45394 | 0.036294 | 1595 |
| SB | Sasa-DAA | Ssa171 | 0.8375 | 0.019865 | 2765 |
| SB | Ssa289 | Ssa171 | 0.48628 | 0.027104 | 387 |

**Table S5.** Pairwise relatedness for all 54 individuals from two populations

| **Population SU** | **LR1** | **LR2** | **LRM** | **QG1** | **QG2** | **QGM** |
| --- | --- | --- | --- | --- | --- | --- |
| **N** | 351 | 351 | 351 | 351 | 351 | 351 |
| **Sum** | -7.275 | -6.225 | -6.750 | -14.220 | -12.780 | -13.500 |
| **Mean** | -0.021 | -0.018 | -0.019 | -0.041 | -0.036 | -0.038 |
| **Median** | -0.029 | -0.027 | -0.029 | -0.059 | -0.047 | -0.055 |
| **SD** | 0.049 | 0.045 | 0.046 | 0.141 | 0.144 | 0.136 |
| **SE** | 0.003 | 0.002 | 0.002 | 0.008 | 0.008 | 0.007 |
| **Min** | -0.129 | -0.120 | -0.105 | -0.446 | -0.428 | -0.332 |
| **Max** | 0.157 | 0.185 | 0.171 | 0.401 | 0.409 | 0.393 |
|  |  |  |  |  |  |  |
|  |  |  |  |  |  |  |
| **Population SB** | **LR1** | **LR2** | **LRM** | **QG1** | **QG2** | **QGM** |
| **N** | 351 | 351 | 351 | 351 | 351 | 351 |
| **Sum** | -6.595 | -6.905 | -6.750 | -12.567 | -14.433 | -13.500 |
| **Mean** | -0.019 | -0.020 | -0.019 | -0.036 | -0.041 | -0.038 |
| **Median** | -0.026 | -0.026 | -0.028 | -0.064 | -0.062 | -0.065 |
| **SD** | 0.047 | 0.044 | 0.044 | 0.147 | 0.153 | 0.144 |
| **SE** | 0.002 | 0.002 | 0.002 | 0.008 | 0.008 | 0.008 |
| **Min** | -0.110 | -0.117 | -0.098 | -0.350 | -0.413 | -0.313 |
| **Max** | 0.308 | 0.217 | 0.235 | 0.582 | 0.620 | 0.601 |

**Table S6.** Generalised linear modelling of sea lice abundance as a function of salmon standard length (L), site of origin (Pop) and individual homozygosity by locus (HL) for (a) all loci and (b) MHC-linked loci showing support for best five models.

1. **All loci**

mfull_all<-glm(Sealice~HL_all*Length*Population,family=poisson,data=Sealice_counts_final)

options(na.action=na.fail)

| Model No. | Intercept | HL | L | Pop | HL:L | HL:Pop | L:Pop | HL:L:Pop | df | AICc | ∆ AICc | Weight |
| --- | --- | --- | --- | --- | --- | --- | --- | --- | --- | --- | --- | --- |
| 5 | 2.317 |  |  | + |  |  |  |  | 2 | 339.3 | 0.00 | 0.234 |
| 39 | 1.569 |  | 0.01283 | + |  |  | + |  | 4 | 339.8 | 0.44 | 0.189 |
| 7 | 1.703 |  | 0.01055 | + |  |  |  |  | 3 | 340.1 | 0.79 | 0.158 |
| 6 | 2.372 | -0.1636 |  | + |  |  |  |  | 3 | 341.4 | 2.08 | 0.083 |
| 40 | 1.615 | -0.0880 | 0.01254 | + |  |  | + |  | 5 | 342.2 | 2.82 | 0.057 |

**Summary best model (m5)**

Estimate Std. Error z value Pr(>|z|)

(Intercept) 2.31729 0.06041 38.36 <2e-16 ***

PopulationSU -1.35045 0.13317 -10.14 <2e-16 ***

Null deviance: 293.21 on 53 degrees of freedom

Residual deviance: 165.69 on 52 degrees of freedom

AIC: 339.1

**Refitted with quasi-poisson (m5)**

Estimate Std. Error t value Pr(>|t|)

(Intercept) 2.3173 0.1078 21.505 < 2e-16 ***

PopulationSU -1.3504 0.2375 -5.685 6.02e-07 ***

(Dispersion parameter for quasipoisson family taken to be 3.181448)

1. **Immune-related loci**

mfull_mhc<-glm(Sealice~HL_mhc*Length*Population,family=poisson,data=Sealice_counts_final)

options(na.action=na.fail)

| Model No. | Intercept | HL | L | Pop | HL:L | HL:Pop | L:Pop | HL:L:Pop | df | AICc | ∆ AICc | Weight |
| --- | --- | --- | --- | --- | --- | --- | --- | --- | --- | --- | --- | --- |
| 32 | 5.5660 | -7.194 | -0.04968 | + | 0.1133 | + |  |  | 6 | 317.2 | 0.00 | 0.444 |
| 64 | 5.4430 | -7.211 | -0.04756 | + | 0.1136 | + | + |  | 7 | 317.3 | 0.13 | 0.416 |
| 128 | 5.3510 | -7.028 | -0.04600 | + | 0.1105 | + | + | + | 8 | 319.5 | 2.32 | 0.139 |
| 6 | 2.5500 | -0.400 |  | + |  |  |  |  | 3 | 333.0 | 15.82 | 0.000 |
| 22 | 2.5930 | -0.480 |  | + |  | + |  |  | 4 | 333.8 | 16.59 | 0.000 |

**Summary best model (m32)**

Estimate Std. Error z value Pr(>|z|)

(Intercept) 5.56623 0.93390 5.960 2.52e-09 ***

HL_mhc -7.19446 1.50726 -4.773 1.81e-06 ***

Length -0.04968 0.01580 -3.144 0.00166 **

PopulationSU -3.45677 0.61710 -5.602 2.12e-08 ***

HL_mhc:Length 0.11333 0.02528 4.484 7.33e-06 ***

HL_mhc:PopulationSU 4.60238 0.99464 4.627 3.71e-06 ***

Null deviance: 293.21 on 53 degrees of freedom

Residual deviance: 133.98 on 48 degrees of freedom

AIC: 315.38

**Refitted with quasi-poisson (m32)**

Estimate Std. Error t value Pr(>|t|)

(Intercept) 5.56623 1.57096 3.543 0.000892 ***

HL_mhc -7.19446 2.53543 -2.838 0.006641 **

Length -0.04968 0.02658 -1.869 0.067682 .

PopulationSU -3.45677 1.03804 -3.330 0.001675 **

HL_mhc:Length 0.11333 0.04252 2.666 0.010439 *

HL_mhc:PopulationSU 4.60238 1.67313 2.751 0.008361 **

(Dispersion parameter for quasipoisson family taken to be 2.829597)

**Re-analysis of model 32 coding HL into three categories (low = 0, moderate = 0.5, high = 1)**

factor32<-glm(Sealice~HL_mhc_type + Length + Population + HL_mhc_type:Length + HL_mhc_type:Population, family = quasipoisson, data = Sealice_counts_final)

Estimate Std. Error t value Pr(>|t|)

(Intercept) 6.14309 1.96100 3.133 0.00304 **

HL_mhc_typeModerate -5.03584 2.34244 -2.150 0.03698 *

HL_mhc_type.High -7.35422 2.71604 -2.708 0.00954 **

Length -0.06152 0.03335 -1.845 0.07165 .

PopulationSU -3.92820 1.29215 -3.040 0.00393 **

HL_mhc_typeModerate:Length 0.08641 0.03994 2.164 0.03584 *

HL_mhc_typeHigh:Length 0.11656 0.04584 2.543 0.01451 *

HL_mhc_typeModerate:PopulationSU 3.39916 1.54642 2.198 0.03313 *

HL_mhc_typeHigh:PopulationSU 4.71507 1.79077 2.633 0.01156 *

(Dispersion parameter for quasipoisson family taken to be 2.858348)

**Table S7**. Sea lice counts and loci data for two sites

| ID | Site | L | W | CF | Sea  lice | HL_all | HL_  neut | HL_  mhc | HL_  mhc_  type | UB  A1 | UB  A2 | UBA  diff | UBA  type | UBA  genotype | DAA  1 | DAA  2 | DAA_  diff | DAA  type | DAA  genotype |
| --- | --- | --- | --- | --- | --- | --- | --- | --- | --- | --- | --- | --- | --- | --- | --- | --- | --- | --- | --- |
| SU01 | SU | 23.5 | 0.205 | 1.579611 | 4 | 0.130000 | 0.154197 | 0.000000 | Low | 134 | 144 | 10 | Het | UBA134144 | 248 | 258 | 10 | Het | DAA248258 |
| SU02 | SU | 22.8 | 0.165 | 1.392129 | 8 | 0.340000 | 0.235811 | 1.000000 | V. High | 140 | 140 | 0 | Hom | UBA140140 | 258 | 258 | 0 | Hom | DAA258258 |
| SU03 | SU | 19.9 | 0.105 | 1.332386 | 9 | 0.420000 | 0.411368 | 0.480864 | Moderate | 140 | 158 | 18 | Het | UBA140158 | 278 | 278 | 0 | Hom | DAA278278 |
| SU04 | SU | 20.6 | 0.145 | 1.658694 | 6 | 0.140000 | 0.159200 | 0.000000 | Low | 134 | 144 | 10 | Het | UBA134144 | 248 | 258 | 10 | Het | DAA248258 |
| SU06 | SU | 22.7 | 0.170 | 1.453354 | 6 | 0.280000 | 0.242812 | 0.519136 | Moderate | 140 | 140 | 0 | Hom | UBA140140 | 258 | 288 | 30 | Het | DAA258288 |
| SU07 | SU | 23.6 | 0.180 | 1.369419 | 3 | 0.220000 | 0.094540 | 1.000000 | V. High | 142 | 142 | 0 | Hom | UBA142142 | 288 | 288 | 0 | Hom | DAA288288 |
| SU08 | SU | 20.0 | 0.105 | 1.312500 | 0 | 0.420000 | 0.327841 | 1.000000 | V. High | 144 | 144 | 0 | Hom | UBA144144 | 258 | 258 | 0 | Hom | DAA258258 |
| SU09 | SU | 21.3 | 0.125 | 1.293514 | 6 | 0.490000 | 0.490951 | 0.480864 | Moderate | 136 | 142 | 6 | Het | UBA136142 | 278 | 278 | 0 | Hom | DAA278278 |
| SU10 | SU | 22.3 | 0.145 | 1.307535 | 3 | 0.230000 | 0.265196 | 0.000000 | Low | 136 | 142 | 6 | Het | UBA136142 | 248 | 258 | 10 | Het | DAA248258 |
| SU11 | SU | 21.0 | 0.140 | 1.511716 | 1 | 0.280000 | 0.327226 | 0.000000 | Low | 134 | 144 | 10 | Het | UBA134144 | 258 | 288 | 30 | Het | DAA258288 |
| SU12 | SU | 23.0 | 0.180 | 1.479412 | 1 | 0.270000 | 0.154197 | 1.000000 | V. High | 134 | 134 | 0 | Hom | UBA134134 | 278 | 278 | 0 | Hom | DAA278278 |
| SU13 | SU | 19.8 | 0.120 | 1.545915 | 2 | 0.350000 | 0.323436 | 0.480864 | Moderate | 134 | 140 | 6 | Het | UBA134140 | 288 | 288 | 0 | Hom | DAA288288 |
| SU14 | SU | 21.0 | 0.115 | 1.241767 | 3 | 0.280000 | 0.243444 | 0.480864 | Moderate | 134 | 146 | 12 | Het | UBA134146 | 258 | 258 | 0 | Hom | DAA258258 |
| SU15 | SU | 22.0 | 0.150 | 1.408715 | 1 | 0.140000 | 0.158653 | 0.000000 | Low | 134 | 144 | 10 | Het | UBA134144 | 248 | 258 | 10 | Het | DAA248258 |
| SU16 | SU | 26.0 | 0.150 | 0.853437 | 1 | 0.430000 | 0.338512 | 1.000000 | V. High | 136 | 136 | 0 | Hom | UBA136136 | 288 | 288 | 0 | Hom | DAA288288 |
| SU17 | SU | 22.0 | 0.120 | 1.126972 | 2 | 0.340000 | 0.313926 | 0.480864 | Moderate | 136 | 144 | 8 | Het | UBA136144 | 208 | 208 | 0 | Hom | DAA208208 |
| SU18 | SU | 19.0 | 0.115 | 1.676629 | 2 | 0.440000 | 0.353060 | 1.000000 | V. High | 136 | 136 | 0 | Hom | UBA136136 | 248 | 248 | 0 | Hom | DAA248248 |
| SU19 | SU | 22.0 | 0.175 | 1.643501 | 3 | 0.290000 | 0.263540 | 0.480864 | Moderate | 134 | 144 | 10 | Het | UBA134144 | 208 | 208 | 0 | Hom | DAA208208 |
| SU20 | SU | 25.0 | 0.155 | 0.992000 | 3 | 0.330000 | 0.311160 | 0.480864 | Moderate | 134 | 144 | 10 | Het | UBA134144 | 248 | 248 | 0 | Hom | DAA248248 |
| SU23 | SU | 21.0 | 0.105 | 1.133787 | 0 | 0.200000 | 0.154197 | 0.519136 | Moderate | 134 | 134 | 0 | Hom | UBA134134 | 258 | 288 | 30 | Het | DAA258288 |
| SU24 | SU | 23.0 | 0.135 | 1.109559 | 1 | 0.430000 | 0.418573 | 0.480864 | Moderate | 134 | 146 | 12 | Het | UBA134146 | 258 | 258 | 0 | Hom | DAA258258 |
| SU25 | SU | 21.0 | 0.105 | 1.133787 | 0 | 0.130000 | 0.071097 | 0.480864 | Moderate | 134 | 160 | 26 | Het | UBA134160 | 278 | 278 | 0 | Hom | DAA278278 |
| SU26 | SU | 25.0 | 0.140 | 0.896000 | 2 | 0.370000 | 0.352001 | 0.480864 | Moderate | 134 | 144 | 10 | Het | UBA134144 | 292 | 292 | 0 | Hom | DAA292292 |
| SU27 | SU | 29.0 | 0.155 | 0.635532 | 0 | 0.210000 | 0.243358 | 0.000000 | Low | 140 | 146 | 6 | Het | UBA140146 | 258 | 288 | 30 | Het | DAA258288 |
| SU28 | SU | 24.0 | 0.150 | 1.085069 | 1 | 0.700000 | 0.651994 | 1.000000 | V. High | 142 | 142 | 0 | Hom | UBA142142 | 248 | 248 | 0 | Hom | DAA248248 |
| SU29 | SU | 20.0 | 0.085 | 1.062500 | 2 | 0.360000 | 0.258196 | 1.000000 | V. High | 138 | 138 | 0 | Hom | UBA138138 | 248 | 248 | 0 | Hom | DAA248248 |
| SU30 | SU | 21.0 | 0.090 | 0.971817 | 1 | 0.340000 | 0.395489 | 0.000000 | Low | 134 | 140 | 6 | Het | UBA134140 | 258 | 288 | 30 | Het | DAA258288 |
| SB01 | SB | 56.0 | 2.390 | 1.360924 | 28 | 0.380000 | 0.437184 | 0.000000 | Low | 140 | 146 | 6 | Het | UBA140146 | 258 | 278 | 20 | Het | DAA258278 |
| SB02 | SB | 61.0 | 3.470 | 1.528762 | 22 | 0.360000 | 0.335883 | 0.519136 | Moderate | 140 | 140 | 0 | Hom | UBA140140 | 228 | 258 | 30 | Het | DAA228258 |
| SB03 | SB | 58.0 | 2.525 | 1.294129 | 14 | 0.080000 | 0.093481 | 0.000000 | Low | 132 | 142 | 10 | Het | UBA132142 | 248 | 258 | 10 | Het | DAA248258 |
| SB04 | SB | 62.0 | 1.640 | 0.688127 | 19 | 0.200000 | 0.160258 | 0.480864 | Moderate | 132 | 144 | 12 | Het | UBA132144 | 248 | 248 | 0 | Hom | DAA248248 |
| SB06 | SB | 52.0 | 2.745 | 1.952236 | 8 | 0.700000 | 0.653907 | 1.000000 | V. High | 144 | 144 | 0 | Hom | UBA144144 | 208 | 208 | 0 | Hom | DAA208208 |
| SB07 | SB | 52.0 | 2.500 | 1.777993 | 11 | 0.210000 | 0.241821 | 0.000000 | Low | 144 | 164 | 20 | Het | UBA144164 | 208 | 248 | 40 | Het | DAA208248 |
| SB08 | SB | 56.0 | 1.995 | 1.136001 | 4 | 0.370000 | 0.265503 | 1.000000 | V. High | 134 | 134 | 0 | Hom | UBA134134 | 258 | 258 | 0 | Hom | DAA258258 |
| SB09 | SB | 68.0 | 2.670 | 0.849150 | 16 | 0.360000 | 0.262310 | 1.000000 | V. High | 132 | 132 | 0 | Hom | UBA132132 | 258 | 258 | 0 | Hom | DAA258258 |
| SB10 | SB | 47.0 | 0.870 | 0.837965 | 14 | 0.220000 | 0.172961 | 0.519136 | Moderate | 140 | 140 | 0 | Hom | UBA140140 | 248 | 258 | 10 | Het | DAA248258 |
| SB11 | SB | 51.0 | 1.640 | 1.236327 | 8 | 0.420000 | 0.326083 | 1.000000 | V. High | 140 | 140 | 0 | Hom | UBA140140 | 258 | 258 | 0 | Hom | DAA258258 |
| SB13 | SB | 60.0 | 3.525 | 1.631944 | 10 | 0.270000 | 0.154197 | 1.000000 | V. High | 144 | 144 | 0 | Hom | UBA144144 | 208 | 208 | 0 | Hom | DAA208208 |
| SB14 | SB | 52.0 | 2.730 | 1.941568 | 2 | 0.280000 | 0.167310 | 1.000000 | V. High | 144 | 144 | 0 | Hom | UBA144144 | 208 | 208 | 0 | Hom | DAA208208 |
| SB15 | SB | 40.0 | 0.790 | 1.234375 | 5 | 0.410000 | 0.396394 | 0.519136 | Moderate | 134 | 134 | 0 | Hom | UBA134134 | 248 | 288 | 40 | Het | DAA248288 |
| SB16 | SB | 50.0 | 0.825 | 0.660000 | 0 | 0.420000 | 0.323624 | 1.000000 | V. High | 140 | 140 | 0 | Hom | UBA140140 | 258 | 258 | 0 | Hom | DAA258258 |
| SB17 | SB | 61.0 | 2.480 | 1.092602 | 4 | 0.360000 | 0.259203 | 1.000000 | V. High | 144 | 144 | 0 | Hom | UBA144144 | 248 | 248 | 0 | Hom | DAA248248 |
| SB18 | SB | 62.0 | 3.060 | 1.283945 | 10 | 0.360000 | 0.338512 | 0.519136 | Moderate | 140 | 140 | 0 | Hom | UBA140140 | 248 | 258 | 10 | Het | DAA248258 |
| SB19 | SB | 65.0 | 3.370 | 1.227128 | 9 | 0.650000 | 0.594949 | 1.000000 | V. High | 134 | 134 | 0 | Hom | UBA134134 | 258 | 258 | 0 | Hom | DAA258258 |
| SB20 | SB | 65.0 | 1.025 | 0.373236 | 6 | 0.230000 | 0.264598 | 0.000000 | Low | 118 | 144 | 26 | Het | UBA118144 | 248 | 258 | 10 | Het | DAA248258 |
| SB21 | SB | 67.0 | 3.095 | 1.029049 | 12 | 0.420000 | 0.415397 | 0.480864 | Moderate | 140 | 144 | 4 | Het | UBA140144 | 288 | 288 | 0 | Hom | DAA288288 |
| SB22 | SB | 62.0 | 3.775 | 1.583952 | 5 | 0.150000 | 0.170059 | 0.000000 | Low | 140 | 144 | 4 | Het | UBA140144 | 208 | 258 | 50 | Het | DAA208258 |
| SB23 | SB | 59.0 | 1.650 | 0.803393 | 6 | 0.480000 | 0.399314 | 1.000000 | V. High | 144 | 144 | 0 | Hom | UBA144144 | 248 | 248 | 0 | Hom | DAA248248 |
| SB24 | SB | 56.0 | 1.505 | 0.856983 | 8 | 0.280000 | 0.247815 | 0.480864 | Moderate | 116 | 144 | 28 | Het | UBA116144 | 258 | 258 | 0 | Hom | DAA258258 |
| SB25 | SB | 57.0 | 1.755 | 0.947660 | 11 | 0.210000 | 0.164663 | 0.519136 | Moderate | 142 | 142 | 0 | Hom | UBA142142 | 258 | 288 | 30 | Het | DAA258288 |
| SB26 | SB | 73.0 | 2.025 | 0.520543 | 7 | 0.140000 | 0.156826 | 0.000000 | Low | 134 | 160 | 26 | Het | UBA134160 | 258 | 288 | 30 | Het | DAA258288 |
| SB27 | SB | 57.0 | 1.800 | 0.971959 | 19 | 0.420000 | 0.329822 | 1.000000 | V. High | 142 | 142 | 0 | Hom | UBA142142 | 208 | 208 | 0 | Hom | DAA208208 |
| SB28 | SB | 56.0 | 2.220 | 1.264122 | 12 | 0.340000 | 0.316555 | 0.480864 | Moderate | 144 | 160 | 16 | Het | UBA144160 | 208 | 208 | 0 | Hom | DAA208208 |
| SB30 | SB | 62.0 | 2.680 | 1.124501 | 4 | 0.290000 | 0.180201 | 1.000000 | V. High | 140 | 140 | 0 | Hom | UBA140140 | 248 | 248 | 0 | Hom | DAA248248 |
